# Discrimination and Health: Results of the Panel ‘Health in Germany’ 2024

**DOI:** 10.25646/13912

**Published:** 2026-03-16

**Authors:** Carmen Koschollek, Marleen Bug, Susanne Bartig, Kathleen Pöge, Caroline Cohrdes, Claudia Hövener, Katja Kajikhina, Jens Hoebel

**Affiliations:** 1 Robert Koch Institute, Department of Epidemiology and Health Monitoring, Berlin, Germany; 2 Freie Universität Berlin, Institute of Sociology, Berlin, Germany; 3 Alice Salomon University of Applied Sciences Berlin, Berlin, Germany; 4 Robert Koch Institute, Department of Infectious Disease Epidemiology, Berlin, Germany

**Keywords:** Discrimination, Health inequalities, Gender identity, Education, Migration, Self-rated general health, Mental health, Prevalence, Social determinants of health, Germany

## Abstract

**Background:**

Discrimination is prohibited by law in specific contexts. Nevertheless, it does occur and may seriously impact health. This contribution analyses social differences regarding the prevalence of experiences of discrimination and its associations with health among adults living in Germany.

**Methods:**

The analyses are based on the population-based panel ‘Health in Germany’ conducted by the Robert Koch Institute. Participants were asked about experiences of everyday discrimination and possible reasons for these experiences. The frequency of experiencing everyday discrimination as well as the occurrence of multiple discrimination were examined for different social groups. Associations between experiences of discrimination and self-rated general as well as mental health were investigated using Poisson regression.

**Results:**

Everyday and multiple discrimination is reported more often by younger, trans or gender diverse persons as well as from people in socioeconomically disadvantaged situations and migrants. The frequency of experiencing everyday and multiple discrimination is associated with progressively worse self-rated general and mental health.

**Conclusions:**

Discrimination is a relevant social determinant of health. The results corroborate the approach of the World Health Organization to reduce and overcome discrimination as a central field of action to foster health equity.

## 1. Introduction

Discrimination can have significant negative impacts on physical and mental health as well as health-related behaviour. Hence, it constitutes a relevant barrier to health equity [1–3]. In many countries, laws are in place that prohibit discrimination [4]. In Germany, the General Act on Equal Treatment (*Allgemeines Gleichbehandlungsgesetz*, AGG) enforces the prohibition of discrimination in specific contexts such as employment and service relationships based on ethnic affiliation or origin, gender or sexual identity, religion or beliefs, age or disability (§§ 1, 2 AGG). However, empirical evidence shows that structural, institutional and interpersonal discrimination exist in Germany [5–7].

In their *World Report on Social Determinants of Health Equity* published in 2025, the World Health Organization (WHO) emphasises the pivotal relevance of structural discrimination as a key determinant of health, and that it needs to be addressed through politics, laws and social norms [4]. Structural discrimination is anchored at the societal level (macro level) and embedded in legislative and economic systems [4, 8]. Institutional discrimination refers to discriminatory routines, processes or practices – including legislative practices – which occur within organisations and institutions, either directly or indirectly, and which systematically (re-)produce barriers to accessing social resources and opportunities for participation (meso level) [9]. Structural and institutional discrimination are mutually dependent and cannot always be distinguished from each other [9, 10]. Interpersonal discrimination occurs between individuals or groups (micro level) and can manifest as exclusion or degradation, or even violence. Discrimination is ‘not solely a matter of individual insults or acts of violence, but a complex system of power within society as a whole, with a long tradition that remains potent to this day and in which we are all entangled’ [11] (authors’ translation).

Discrimination is defined as processes whereby certain social groups or individual members of these groups are disadvantaged by individual, collective or institutional behaviour [5]. From a sociological perspective, discrimination is based on the subdivision and distinction of categories of people or imagined groups, which are not necessarily based on real groups [12]. This distinction between social groups and categories of people is constructed and serves to ‘create, establish, and justify boundaries and hierarchies, in particular power asymmetries, socioeconomic inequalities, and unequal opportunities for recognition’ [12, p. 21, authors’ translation] (structural dominance [13]). Groups constructed in this way are attributed positive or negative characteristics and assigned positions of social power. These characteristics and traits are then assumed to be inherent to the people classified in that group, regardless of whether they actually possess them [5, 10, 12]. The negative characteristics attributed to the group can lead to rejection and disadvantage for the person assigned to it on an individual level [5, 10]. ‘Viewed in this light, discrimination is problematic not only due to its disadvantageous consequences, but also because of the power to impose group affiliations on individuals from which they cannot elude’ [12, p. 28, authors’ translation]. These groups are constructed along different categories or dimensions, generating specific forms of discrimination such as sexism [14], ageism [15], classism [16] and racism [17]. Regardless of whether discrimination is obvious or goes unnoticed, it causes harm to those affected [3, 7]. It can occur along one or more categories of discrimination – for example, age and gender (multidimensional). Moreover, categories can be inseparably combined, generating specific experiences (multiple and intersectional discrimination) [5, 8, 18].

The literature suggests that discrimination impacts the economy, politics and society [8] as well as health [2, 3, 19]. Worse outcomes are described along different categories of discrimination for mental health (e.g. depression, anxiety disorders and reduced well-being) and physical health (e.g. worse self-rated overall health, elevated blood pressure and cardiovascular diseases). Additionally, associations have been reported with lower levels of healthcare utilisation and unfavourable health behaviour (e.g. substance use): for racist discrimination [3, 20–22], discrimination based on gender or sexual identity [3, 23–27], older age [28–30], body weight [31–33] as well as for disability-related discrimination [34–37]. In some studies, researchers have found that discrimination based on the socioeconomic position partly explains social gradients in health and thus mediates the relationship between socioeconomic disadvantage and lower health chances and higher morbidity risks [38, 39]. As a specific form of stress, discrimination shows direct associations with mental health, such as reduced well-being or even depressive symptoms [2, 21]. In addition, social withdrawal accompanied by a loss of social networks contributes to worse mental health outcomes [7]. Possible strategies for dealing with experiences of discrimination – for example, unfavourable health behaviour such as substance use – might in the long run contribute to additional negative impacts on physical health [3, 7, 21]. At the same time, discrimination can lead to higher levels of certain biological stress markers (e.g. cortisol) and thus affect physical and mental health. The interplay of various of these stress markers in response to chronic stress, resulting in physiological dysregulation of the organism, is referred to as allostatic load [40]. This leads to a long-term wear and tear of the organism, accompanied by a variety of physical illnesses [7] and increased mortality throughout the life course [41].

**Key messages**

► More than two thirds of the adult population report experiencing discrimination in their everyday life rarely or sometimes; 12.2 % indicate such experiences occur often or very often.
► More than half report a single reason for their experiences of discrimination, while 24.3 % indicate two reasons and 22.5 % three or more reasons.
► For multiple discrimination, the most common combinations of characteristics are age and gender followed by age and socioeconomic position as well as socioeconomic position and origin.
► For everyday as well as multiple discrimination, there are considerable social differences based on age, gender, socioeconomic position, and migration-related indicators.
► Experiencing everyday as well as multiple discrimination is associated with progressively worse self-rated general and mental health.

Intersectional discrimination, where different categories of discrimination overlap and intertwine, is also relevant because it can have distinct negative health consequences – especially, if it is experienced frequently [42]. In recent years, efforts have been made to capture the concept of intersectionality using quantitative data, with the aim of allowing the associations between multiple discrimination and health inequalities to be measured [43, 44]. In a systematic review published in 2021 that analysed 65 studies, intersectional discrimination was associated with differences in mental and physical health as well as health-related behaviour [42]. At the same time, substantial theoretical and methodological challenges were identified in relation to, for example, the selection of relevant categories and the adequate statistical representation of their interdependencies, rather than considering them as only additive [42–44]. Additionally, a lack of longitudinal data and insufficient consideration of structural and contextual factors impede comprehensive analyses of the cumulative effects of discrimination [13, 42, 44]. As these challenges remain unresolved, we do not claim to capture intersectional discrimination in the present contribution. We speak of multiple discrimination when, according to the respondents’ self-reports, there are two or more reasons for the experienced discrimination, and these have a cumulative effect on health in the sense that they reinforce each other.

So far, in Germany there have been few quantitative analyses of the relationship between discrimination and health; large, population-based studies are especially scarce. The available analyses have focused on discrimination in the healthcare sector [45, 46], or specific population groups such as younger [47] or older [48] people, migrants [49–53] or LGBTIQ* people [54, 55]. As a large, representative panel survey, the Socio-Economic Panel (SOEP) captured detailed data on discrimination in 2022. However, analyses are still pending [56]. Against this background, the aim of this contribution is to describe the frequency of self-reported experiences of discrimination in everyday life among adults in Germany according to demographic, socioeconomic and migration-related characteristics, based on population-based data from the Robert Koch Institute (RKI). Additionally, this contribution focuses on different categories of discrimination, as well as possible experiences of multiple discrimination. Associations between the frequency of experiences of discrimination in everyday life, considering multiple discrimination and different discrimination categories, and the health status are analysed using nationwide data from Germany.

## 2. Methods

### 2.1 Study design and sample

The analyses are based on the population-based panel ‘Health in Germany’, which was established with a recruitment study in 2024 by the RKI. Sampling was based on a double-stratified random selection: 359 primary sampling units (sample points) were randomly drawn from all municipalities in Germany, considering the regional structure (the first selection stage). In the second selection stage, addresses were drawn for each sample point, stratified by age group, from the address registers of the respective residents’ registration offices. The selected individuals were invited to take part in a short survey and asked for their consent to participate in future surveys as part of the panel [57].

**RKI Panel ‘Health in Germany’ 2024**

**Data holder:** Robert Koch Institute

**Objectives:** To provide comprehensive data on the health status, health-related behaviour and health care of the population in Germany, with the future possibility of longitudinal comparisons and analysis of trends over time

**Study design:** Panel study with a mixed-mode approach (online and written-postal participation)

**Population:** German-speaking population aged 18 and over in private households with main residence in Germany

**Sample:** Probabilistic/representative sample of the ‘Health in Germany’ panel infrastructure

**Participants in the 2024 annual wave:** A total of 41,376 of the persons registered in the panel took part in at least one of the three sub-waves in 2024.

Questionnaire A: 14,759 women, 12,374 men, 66 persons with other gender identities

Questionnaire B: 14,828 women, 12,258 men, 61 persons with other gender identities

Questionnaire C: 14,709 women, 12,329 men, 64 persons with other gender identities

Questionnaire D: 14,872 women, 12,368 men, 66 persons with other gender identities

**Data collection:**

1st sub-wave: 28.05.2024 – 05.08.2024

2nd sub-wave: 12.08.2024 – 14.10.2024

3rd sub-wave: 28.10.2024 – 06.01.2025

More information at www.rki.de/panel-en

For the first annual wave in 2024, the RKI panel comprised 46,863 registered participants aged 18 years and older: 24,881 women, 21,856 men and 126 people with another gender identity. In 2024, they were invited to participate in health surveys at three points in time (sub-waves). In each sub-wave, they received one of four questionnaires, each covering different health topics. In the first sub-wave, the questionnaire also included in-depth questions on sociodemographic characteristics. Participation was possible web-based and in writing by post. Data collection started in May 2024 with the first sub-wave and ended at the beginning of January 2025 with the third sub-wave [58]. The response rate (the proportion of participants relative to the people registered in the panel) was 75 % – 81 % in the individual sub-waves according to the standards of the American Association for Public Opinion Research (AAPOR) [59]. A detailed description of the methodology can be found elsewhere [58].

### 2.2 Indicators and operationalisation

Self-reported discrimination in everyday life was captured using an adapted version of the Everyday Discrimination Scale (Short Version) [60, 61]. In the first stage, the instrument captured the frequency of experiences of interpersonal discrimination in five everyday situations (e.g. ‘You receive poorer service than other people (e.g. at restaurants or stores).’) with the answer options ‘very often’, ‘often’, ‘sometimes’, ‘rarely’ and ‘never’. For the analyses, answers were compiled into (1) ‘often/very often’ if at least one item was answered with often or very often, and (2) ‘rarely/sometimes’ if at least once rarely or sometimes was answered and often or very often was not reported at the same time. These two categories were contrasted with those who answered (3) ‘never’ for all five items. Among the five items, two missing values were tolerated; if there were three or more missing values, then the categorised variable was set to missing overall. In the second stage, the instrument captured possible reasons (in the sense of (externally) ascribed social categories) why this discrimination has occurred from the point of view of the respondents (e.g. ‘gender’, ‘origin, accent, language, appearance, name’, ‘sexual orientation’). For respondents reporting having ever experienced discrimination, the answer options on the reasons for experiencing discrimination were collapsed into (1) one reason, (2) two reasons and (3) three or more reasons reported (multiple discrimination). Additionally, all ten offered reasons were combined to analyse which combinations occurred most often.

Demographic (gender, age), socioeconomic (educational level, income occupational status, experiences of unemployment) and migration-related (country of birth, citizenship, German language proficiency) variables were selected as stratification variables. Gender was captured in a two-staged approach [62]. For the first stage, respondents were asked about their sex stated on their birth certificate (‘female’, ‘male’). For the second stage, they were asked to which gender they feel they belong (‘female’, ‘male’, ‘another one, that is…). For the variable gender, respondents were categorised as cisgender if the sex on their birth certificate matched their answers to their gender identity (e.g. ‘female’ and ‘female’ categorised as cis female). Respondents whose assigned sex at birth and gender identity did not correspond were included in the category trans/gender diverse [26] due to a low number of cases. This applies to female and male trans people as well as those who do not identify as female or male but state a gender identity within the non-binary spectrum. Trans women and men were integrated into the trans/gender diverse category despite their binary gender identity, as it can be assumed that, in contrast to cisgender people, they probably experience discrimination more often. The respondents’ age was categorised as 18 – 29 years, 30 – 44 years, 45 – 64 years, and 65 years and older.

Responses on the highest educational and vocational qualifications were compiled according to the Comparative Analysis of Social Mobility in Industrial Nations (CASMIN) classification into low, medium and high formal education [63]. The household net income was captured by the question ‘What is the total monthly net income of your household?’, where respondents could either answer with an actual amount or in income categories. Considering the number and age of all household members, the equivalised net income was calculated [64] and categorised into ‘< 60 % of the median’ (poverty risk threshold), ‘60 % – 150 % of the median’ and ‘> 150 % of the median’. Missing values on income were imputed using regression analyses [65]. The occupational status was captured by the question ‘Which of the following most accurately describes your current life situation?’. The answer options ‘I work full time’ and ‘I work part-time’ were combined into ‘employed’, and the answer options ‘I am currently unemployed’, ‘I am retired/partially retired’ and ‘I am not employed for other reasons (e.g. students, voluntary social year, househusband/housewife)’ were summarised as ‘not employed’. In terms of the occupational status, only respondents at an employable age (18 – 64 years) were considered. Experiences of unemployment were surveyed by the question ‘Have you ever been unemployed in the last 5 years?’, and the answer options ‘yes’ and ‘no’ were contrasted.

Country of birth was captured by the question ‘In which country were you born?’. Respondents born in Germany were contrasted to those born abroad. Citizenship (‘What citizenship(s) do you have?’) was differentiated into ‘German citizenship’ and ‘another citizenship’. German language proficiency was captured in two steps. First, the native language was surveyed (‘German’, ‘another language’). Those not stating German as their native language were asked to self-rate their German language proficiency (‘How would you rate your German language proficiency?’). Answers to both questions were categorised into ‘native language/very good/good’ and ‘moderate/poor/very poor’.

Self-rated general health (‘How is your health in general?’) and self-rated mental health (‘How would you describe your mental health in general?’) were selected as indicators for the health status. For self-rated general health, answers were categorised as ‘fair’, ‘poor’, and ‘very poor’ (= 1) and contrasted to ‘good’ and ‘very good’ (= 0) [66]. The five-point scale for self-rated mental health was categorised analogously to other public health surveillance systems, such as the Canadian ‘Positive Mental Health Surveillance Indicator Framework’ [67], and summarised into ‘good’, ‘fair’, and ‘poor’ (= 1) and contrasted to ‘very good’ and ‘excellent’ (= 0).

### 2.3 Statistical methods

For the analyses, first, information from the recruitment study (dataset version (DV) 2), the questionnaire on mental health (DV 5) as well as the in-depth information on sociodemographic characteristics (DV 5) were combined. Respondents were included if they were (a) 18 years and older at the time of the recruitment study and (b) had answered at least three questions on the first stage of the instrument to capture everyday discrimination.

The prevalence and 95 % confidence intervals (95 % CI) for self-reported experiences of everyday discrimination were calculated at the item level as well as categorised as ‘rarely/ sometimes’ and ‘often/very often’; and stratified by demographic, socioeconomic and migration-related characteristics. Chi-squared tests were performed to analyse possible group differences. The reasons for the experienced discrimination were analysed descriptively as proportions with 95 % CI; differences by gender were also examined (a stratification for trans/gender diverse was not possible due to the small number of cases). Additionally, the categorial number of stated reasons (multiple discrimination) was stratified by demographic, socioeconomic and migration-related characteristics, and chi-squared tests were used to examine group differences. A p-value < 0.05 was considered to indicate a statistically significant difference.

To examine associations between discrimination and the self-rated general and mental health, the prevalence of both health outcomes along with 95 % CI were initially stratified by the frequency of experiences of everyday discrimination and the occurrence of multiple discrimination. Afterwards, answers on the frequency of everyday discrimination and the categorical number of reasons were combined into one variable (e.g. ‘rarely/sometimes, one reason’) and analysed for associations with both health outcomes in Poisson regression models. First, univariable analyses were conducted, followed by successively controlling for demographic (model 1), socioeconomic (model 2, except for the employment status due to the restriction to employable age), and migration-related characteristics (model 3). The aim was to examine the impact of discrimination while holding all other factors constant. The prevalence ratios (PR) and their 95 % CI are presented with forest plots. Statistically significant associations were assumed if the 95 % CI did not include the value 1. A PR larger than 1 means that the prevalence of a worse self-rated general and mental health, respectively, is increased compared to the reference group without experiences of discrimination.

A weighting factor was included in the analyses to correct deviations of the sample from the population structure in Germany due to selective participation. The multi-stage sample weight considers the sample weight of the recruitment study as well as a drop-out weight to account for selective participation in the sub-waves throughout 2024. The adjustment weight adjusts the sample to population figures as of 31 December 2023 and the Microcensus 2021 in terms of age, gender, BIK municipality size [68], federal state, educational level (CASMIN [63]) and household size (single vs multi-person households). To account for clustering of participants within sample points and to adequately integrate weighting when calculating the 95 % CI and p-values, all analyses were conducted using survey procedures for complex samples. All analyses were conducted using Stata 17.0 (Stata Corp., College Station, TX, USA).

## 3. Results

Overall, 26,645 respondents (14,412 cis women (50.7 %; 95 % CI: 49.9 – 51.5), 12,086 cis men (48.6 %; 95 % CI: 47.8 – 49.4) – henceforth women and men, respectively – and 147 trans/gender diverse people (0.7 %; 95 % CI: 0.5 – 0.8)) were included in the following analyses. A description of the study population according to the above-mentioned characteristics can be found in Annex Table 1.

### 3.1 Self-reported discrimination in everyday life

**Figure 1:**
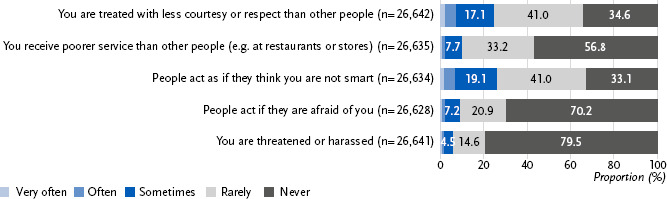
Frequency of experiencing discrimination in everyday life situations. Source: Panel ‘Health in Germany’ 2024

More than two thirds (69.2 %; 95 % CI: 68.4 – 69.9) of the adult population reported experiencing discrimination in their everyday life rarely or sometimes; 12.2 % (95 % CI: 11.6 – 12.9) reported that such experiences occurred often or very often. Overall, 18.6 % (95 % CI: 18.0 – 19.3) reported they did not experience any discrimination in their everyday life. The most commonly reported form of discrimination was not being taken seriously, followed by being treated with less courtesy or respect than other people (Figure 1). The least frequently reported experience was being threatened or harassed, although one in five respondents reported experiencing this at least rarely.

Compared to cisgender people, trans and gender diverse people reported experiencing everyday discrimination far more often (Table 1). Among cisgender people, for the younger age groups (18 – 29 years (p < 0.001) and 30 – 44 years (p = 0.035)), discrimination was reported more often by women. Additionally, there was an age gradient in reporting experiencing discrimination often or very often to the disadvantage of younger age groups. The prevalence of experiencing discrimination was much higher in the group with a low level of formal education compared to the group with a high level of formal education. There were considerable differences according to income: the proportion of those who reported experiencing discrimination often or very often was more than twice as high in the group at risk of poverty in comparison to those in the high income group. Additionally, those currently not employed as well as those reporting experiences of unemployment within the last five years more frequently experienced discrimination often or very often. According to the migration-related characteristics, those not born in Germany, without German citizenship and lower levels of German language proficiency more frequently experienced discrimination often or very often.

### 3.2 Reasons for the experienced discrimination

**Figure 2:**
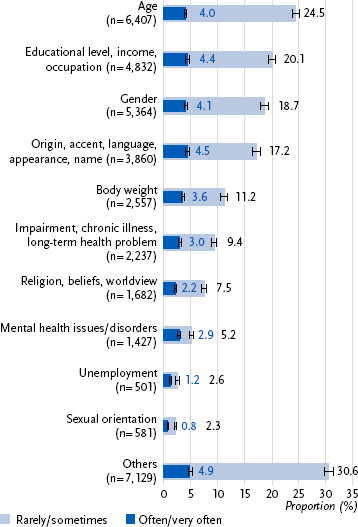
Prevalence of the reported reasons for the experienced discrimination differentiated for rarely/sometimes (light blue) and often/very often (dark blue) reported discrimination (n = 11,034 women, n = 8,871 men and n = 128 trans/gender diverse persons). Source: Panel ‘Health in Germany’ 2024

For those with experiences of discrimination in everyday life, more than a quarter stated it was due to age (28.5 %); 24.5 % reported they experienced discrimination due to age rarely or sometimes, and 4.0 % stated they experienced discrimination due to age often or very often (Figure 2). Another quarter stated they experienced discrimination because of their educational level, income or occupation; this was stated more often by men compared to women (29.0 %; 95 % CI: 27.7 – 30.4 vs. 20.1 %; 95 % CI: 19.1 – 21.2). Both categories, gender as well as origin, accent, language, appearance or name, were overall stated by one in five as reasons why they experienced discrimination. Gender was stated more often by women (34.4 %; 95 % CI: 32.9 – 35.9 vs. 10.6 %; 95 % CI: 9.8 – 11.6), while origin, accent, language, appearance or name was stated more often by men (25.4 %; 95 % CI: 24.0 – 26.7 vs. 18.0 %; 95 % CI: 16.8 – 19.1). One in seven reported body weight as a reason for being discriminated against; it was reported more frequently by women than men (15.6 %; 95 % CI: 14.6 – 16.6 vs. 13.7 %; 95 % CI: 12.6 – 14.8 [p = 0.009]). Discrimination because of an impairment, chronic illness or longstanding health problem was reported by one in eight respondents who had experienced discrimination. Nearly one in ten stated that they had been discriminated against because of their religion, beliefs or worldview; this was reported less frequently by women compared to men (8.7 %; 95 % CI: 7.8 – 9.6 vs. 10.8 %; 95 % CI: 9.9 – 11.8). In contrast, the categories mental health issues or disorders, unemployment and sexual orientation were reported less often as reasons for everyday discrimination. However, most often other, unspecified reasons for the experienced discrimination were stated: more than a third reported this, men more often than women (38.1 %; 95 % CI: 36.7 – 39.4 vs. 33.1 %; 95 % CI: 32.0 – 34.4).

### 3.3 Experiences of multiple discrimination

**Figure 3:**
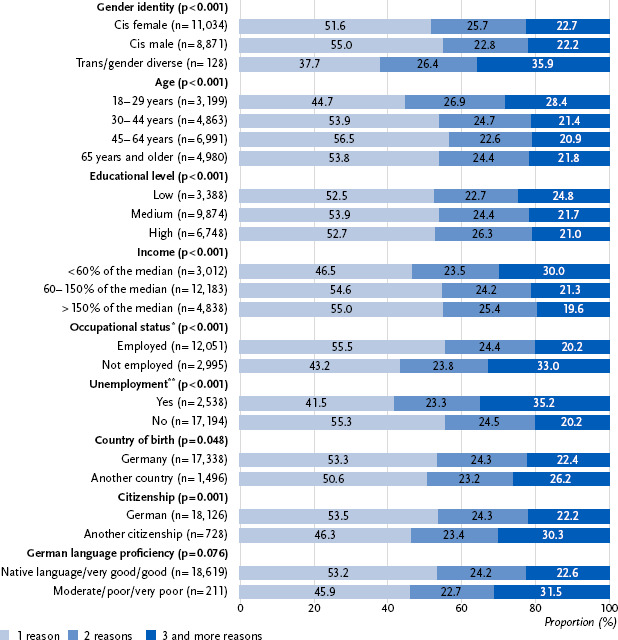
Prevalence of multiple discrimination stratified by the demographic, socioeconomic and migration-related characteristics, in percentages. Source: Panel ‘Health in Germany’ 2024 ^*^employable age, 18 – 64 years ^**^within the last five years

Across all presented categories of discrimination, often not only one reason for having been discriminated against was mentioned. For example, 78.1 % (95 % CI: 76.7 – 79.4) of those who stated experiencing discrimination because of their age also stated that they had been discriminated against for one or more additional reasons. There were similar results for educational level, income and occupation (75.4 %; 95 % CI: 73.7 – 77.1); gender (79.9 %; 95 % CI: 78.5 – 81.3); and origin, accent, language, appearance and name (79.1 %; 95 % CI: 77.1 – 81.1). When considering the total proportion of those who stated they had been discriminated against for more than one reason (46.8 %; 95 % CI: 45.8 – 47.8) compared to those who reported only one reason (53.2 %; 95 % CI: 52.2 – 54.2), there were notable social differences. Trans and gender diverse people reported multiple discrimination more frequently than cisgender people (Figure 3). Among cisgender people, multiple discrimination was reported more often by women in the youngest age group (18 – 29 years, p < 0.001); however, it was reported more often by men in the oldest age group (65 years and older, p = 0.033). Overall, an age gradient can be observed to the disadvantage of the youngest age group (18 – 29 years). Prevalence also differed in terms of socioeconomic characteristics. Especially people currently unemployed and those who have experienced unemployment within the last five years reported multiple reasons for being discriminated against. Additionally, there were observable differences for the migration-related characteristics to the disadvantages of those not being born in Germany and having no German citizenship. Moreover, the proportion of those with lower levels of German language proficiency reporting multiple discrimination was slightly higher.

**Figure 4:**
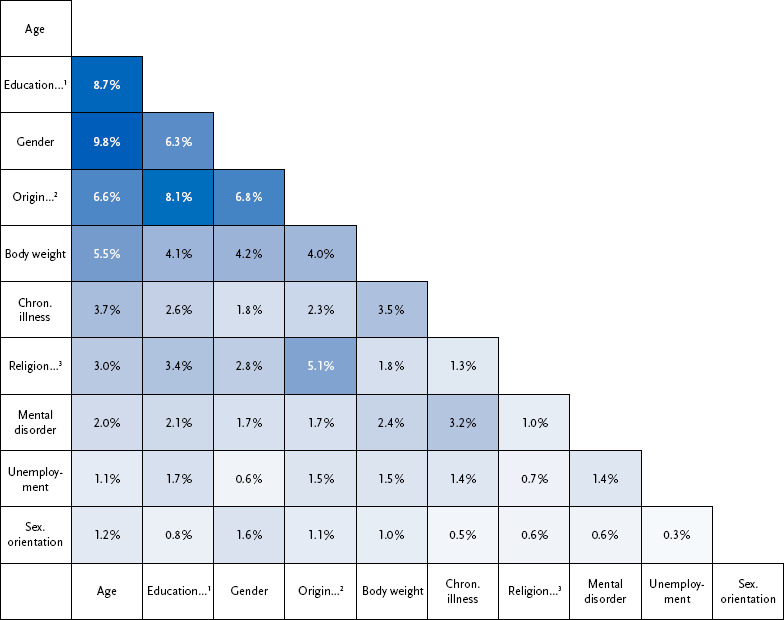
Frequency of reported combinations of reasons for experiencing discrimination in everyday life (n = 11,034 women, n = 8,871 men and n = 128 trans/gender diverse persons). Source: Panel ‘Health in Germany’ 2024 The shading of the boxes corresponds to the frequency of reporting; the darker the shading, the more often the respective combination was stated. For better readability, the denomination of the categories is shortened in the figure and in the text. The proper denomination of the categories can be found in Figure 2. ^1^Educational level, income, occupation ^2^Origin, accent, language, appearance, name ^3^Religion, beliefs, worldview

When combining the reported reasons for having experienced discrimination in everyday life, nearly one in ten reported being discriminated against because of their age and gender, followed by the combination of educational level and age and the combination of educational level and origin (Figure 4). Additionally, about one in fifteen stated origin and gender, origin and age as well as educational level and gender as reasons for discrimination. The combinations age and body weight as well as origin and religion were reported by approximately one in twenty. Other combinations of reasons were less frequent.

### 3.4 Discrimination and health

There is a clear gradient when stratifying the prevalence of a fair to very poor self-rated general health by the frequency of experiences of discrimination in everyday life. Of those who never experienced discrimination, 30.1 % (95 % CI: 28.3 – 31.8) reported a fair to very poor self-rated general health, in contrast to 34.6 % (95 % CI: 33.5 – 35.7) of those who experienced discrimination rarely or sometimes and 46.8 % (95 % CI: 44.1 – 49.7) of those who experienced discrimination in their everyday life often or very often. There is a comparable gradient regarding self-rated mental health: while 55.5 % (95 % CI: 53.6 – 57.3) of those without experiences of discrimination rated their mental health as good, fair or poor, the respective prevalence was higher for those who experienced discrimination rarely or sometimes (66.0 %; 95 % CI: 65.1 – 66.9) or often or very often (77.9 %; 95 % CI: 75.6 –80.1). Multiple discrimination was also associated with health: the prevalence of rather poor self-rated general health for those who reported a single reason for their experiences of discrimination was 31.2 % (95 % CI: 29.9 – 32.5), but it was higher if multiple reasons were indicated (2 reasons: 36.8 %; 95 % CI: 34.8 – 38.9; 3 and more reasons: 44.9 %; 95 % CI: 42.7 – 47.1). There were comparable results for respondents who rated their mental health as good, fair or poor: the prevalence for those who reported a single reason for their experiences of discrimination was 64.4 % (95 % CI: 63.0 – 65.7) and notably higher if multiple reasons were indicated (2 reasons: 68.7 %; 95 % CI: 66.9 – 70.4; 3 and more reasons: 72.6 %; 95 % CI: 70.8 – 74.3).

**Figure 5:**
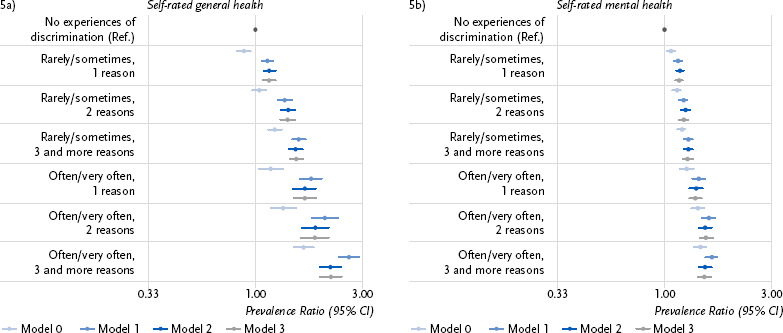
Prevalence ratios for a fair to very poor self-rated general health (5a) and for good, fair or poor self-rated mental health (5b) by the frequency of experiencing discrimination and number of indicated reasons. Source: Panel ‘Health in Germany’ 2024 CI = Confidence Interval 5a: Model 0: n = 26,622 – Null model Model 1: n = 26,622 – M0 + Gender identity and age (metric) Model 2: n = 25,890 – M1 + Education, income, experience of unemployment within the last 5 years Model 3: n = 24,282 – M2 + Country of birth, citizenship, German language proficiency 5b: Model 0: n = 26,607 – Null model Model 1: n = 26,607 – M0 + Gender identity and age (metric) Model 2: n = 25,882 – M1 + Education, income, experience of unemployment within the last 5 years Model 3: n = 24,275 – M2 + Country of birth, citizenship, German language proficiency

For the most part, these gradients remain unchanged when combining the frequency of experiencing discrimination in everyday life as well as the number of reasons for experiencing it into one variable, and the associations with self-rated general and mental health are analysed using Poisson regression models (Figure 5). For self-rated general health (Figure 5a), the PRs increase as the frequency of experiencing discrimination and the number of indicated reasons increase throughout all models (except for the null model (light blue)). This holds true if the models are statistically controlled for demographic (M1, medium blue), socioeconomic (M2, dark blue) and migration-related characteristics (M3, grey). The PRs are increased compared to the reference group, who never experienced discrimination. The same pattern can be observed for self-rated mental health (Figure 5b). In model 3, these results are also observable for gender-stratified analyses – for self-rated general health (Annex Table 2) and self-rated mental health (Annex Table 3).

## 4. Discussion

Experiences of discrimination are widespread in Germany: more than two thirds of the adult population reported experiencing discrimination at least rarely or sometimes within their everyday life, while one in eight reported that such experiences occured often or very often. Multiple discrimination is also prevalent, with half of those who experienced discrimination reporting two or more reasons for these experiences. Trans and gender diverse people, younger age groups and people with lower levels of education, income and without employment reported everyday discrimination, and multiple discrimination disproportionately more often. The migration-related characteristics country of birth, citizenship and German language proficiency also proved relevant for both the frequency of everyday discrimination and for multiple discrimination. This study analysed the way in which discrimination regarding different discrimination categories is associated with health. The results show a distinct and statistically reliable correlation between experiences of discrimination and the health status: the more often discrimination is experienced, and the more reasons the person perceives for these experiences, the worse one rates their own health status. The associations between multiple discrimination and health status hint at the need to increase consideration of an intersectional perspective in future analyses.

Age was the most often mentioned reasons for the experienced discrimination, which is in line with the results of the Federal Anti-Discrimination Agency (*Antidiskriminierungsstelle des Bundes*, ADS) as well as with results of the European Social Survey (ESS): in both studies age was the most frequently mentioned discrimination category [5, 69]. That discrimination is more often perceived by younger age groups is consistent with the ADS [5] and the ESS [69] results. At the same time, the ADS results showed that more than one in six respondents aged 60 years and older perceived discrimination because of their advanced age. Differentiated analyses of the German Aging Survey 2023 substantiate this finding: 7.7 % of participants indicated discrimination due to their advanced age [48]. Given that there has been much more research attention dedicated to discrimination based on older age [70], discrimination against younger people represents an important field of research, which should also focus on discrimination in digital spaces in the form of cyberbullying or hate speech. Furthermore, discrimination in everyday life and multiple discrimination were reported more frequently by young women compared to young men. This finding is in line with a study from the German Youth Institute (*Deutsches Jugendinstitut*, DJI) [47]. It is especially important because within recent years, the prevalence of symptoms of depression and anxiety has increased, especially among young women [71], and studies have shown that discrimination is associated with increased risks of psychological distress [3, 21]. However, in light of demographic ageing, discrimination in old age should not be forgotten, as the health status worsens with increasing age – and especially older people with health impairments experience discrimination in their everyday lives [48]. Additional research attention should be placed on the intersections between ageism and other discrimination categories because the results presented as well as the ADS results (78.7 %) show that the majority of people who perceive discrimination because of their age also report discrimination for other reasons [5]. According to our results, combinations of the categories age and gender as well as age and socioeconomic position seem particular relevant.

The socioeconomic position (educational level, income and occupation) was the second most often stated reason for perceived discrimination, a finding that is also in line with the ADS results [5]. Within the SOEP innovation sample, the socioeconomic position also proved to be a relevant discrimination category [72], and other studies have shown that people experiencing poverty perceive discrimination more frequently [73]. People in a disadvantaged socioeconomic position and those experiencing unemployment reported experiencing discrimination as well as multiple discrimination more frequently. Discrimination due to one’s socioeconomic position often goes along with reporting discrimination for other reasons, as shown by our results as well as the ADS (74.2 %). One relevant additional category in our study is origin, accent, language, appearance and name, which might be explained by the socioeconomic discrimination of migrants [74, 75]. Furthermore, discrimination due to the socioeconomic position was reported more often by cis men than cis women, a finding that may hint at socially established norms [76], although this requires further investigation. However, due to the widespread occurrence of discrimination based on the socioeconomic position, the ADS had already recommended in 2017 to include the socioeconomic position as an additional attribute to be protected by the General Act on Equal Treatment (*Allgemeines Gleichbehandlungsgesetz*) [5]. In the future, there is a need to investigate the extent to which the relationship between the socioeconomic position and the health status [77] may be explained by experiences of discrimination, to determine how much discrimination contributes to health inequity. Precarious socioeconomic situations such as poverty and unemployment are ascribed a social stigma, which is perceived by those affected by everyday experiences of social exclusion and marginalisation and, hence, might affect their health and well-being [78, 79]. Study results show that only expecting to be stigmatised because of poverty or unemployment is associated with reduced well-being and reduced health satisfaction [80, 81]. In Germany, people experiencing poverty face discrimination in many areas of life and within society, particularly in the housing market, leisure and culture, the educational system, mobility, political and societal discourses, the labour market, administrative offices and authorities, and within the health system [6, 73]

The third most often stated category of discrimination was gender. This, again, is in line with the ADS [5] and ESS [69] results. In the analyses from the present study, the vast majority of those who stated gender as a reason for their experiences of discrimination also stated they were discriminated against for one or more further reasons; the proportion was higher than that in the ADS study (56.3 %) [5]. Trans and gender diverse people most often reported experiences of everyday and multiple discrimination. Additionally, the analyses revealed worse health outcomes for both self-rated general and mental health for trans and gender diverse people. The binary gender and cis-normative system is deeply rooted in society and can lead to multiple discrimination on the structural, institutional and interpersonal level, to disadvantages and even violence against trans and non-binary people [26, 55, 82]. Societal norms of heterosexuality may lead to comparable experiences among people who do not identify themselves as heterosexual [82]. These experiences can cause chronic stress and negatively impact physical and mental health as well as health-related behaviour [26, 83–85]. An additional gender-related aspect is that compared to cis men, cis women stated more frequently that their experiences of discrimination were due to gender. This may indicate an increased perception of discrimination [86], but it also highlights the various gender hierarchies embedded in different societal levels, which can result in increased discrimination, disadvantages and violence against women [87, 88]. Furthermore, compared to cis men, cis women more often reported body weight as a reason for their experiences of discrimination, a finding consistent with a study among young adults conducted by the DJI [47]. This refers to societal norms of beauty that promote a lean physique, particularly for women. These norms are closely linked to discrimination based on (higher) body weight as well as gender-related inequities [89, 90]. Body weight was the fifth most often stated reason for discrimination in the present study.

The fourth most often reported reason for experiencing discrimination was origin, accent, language, appearance and name, which might refer to experiences of racism. Racist discrimination was the third most often mentioned discrimination category within the ESS results [69]; in the ADS results, racist discrimination followed the category religion and worldview [5]. The latter was mentioned less often in the present study, but it was often stated in combination with the category origin. The migration-related characteristics country of birth, citizenship and German language proficiency played a role regarding experiences of everyday and multiple discrimination. For discrimination itself, there were comparable results in the SOEP innovation sample [72]. The results of the National Monitoring of Discrimination and Racism (*Nationaler Diskriminierungs- und Rassismusmonitor*, NaDiRa) of the German Centre for Integration and Migration Research (*Deutsches Zentrum für Integrations- und Migrationsforschung*, DeZIM) show that people who are ascribed racist attributes disproportionally often experience discrimination [52]. Additionally, these results show that in particular, people who identify as Muslim mention religion as a reason for experiencing discrimination [52]. Moreover, people who are ascribed racist attributes are particular likely to experience discrimination in institutional areas of life, at administrative offices and authorities, at the police and judiciary as well as in public and leisure [52]. The health interview survey GEDA Fokus, conducted by the RKI among people with selected citizenships, showed that discrimination is significantly associated with various health outcomes among migrants [49, 50, 91].

Overall, one in five of the adult population in Germany reported experiencing discrimination based on impairments or chronic illnesses and mental health problems or disorders. Both categories were comparatively often reported in combination. The category disability/impairment was also relevant in the ADS results [5]. However, it remains unclear whether people would have subsumed mental health issues under this category. Because we did not stratify by health-related indicators, we cannot determine whether in our sample, people with poorer health reported experiences of discrimination more often. Spuling et al. [48] suggest such associations, so it would be important to examine this issue in future work. According to the panel ‘Health in Germany’ 2024, nearly half of the adult population reported chronic illnesses, and depressive symptoms can be assumed in about one in five [92]. Against this background, experiences of discrimination may further exacerbate these health problems.

It is necessary to highlight that the ‘others’ category was stated most often as a reason for experiencing discrimination. The ADS study included a similar category, but with an additional open-ended text field. Here, reasons were mentioned that would likely have been represented across several of the categories used in our study. Additionally, the ADS study identified family situation as a reason for discrimination, for example, among single parents or people with many children [5]. In the future, it will be investigated whether such an open-ended response option should also be included in the RKI panel, also for ethical reasons, to allow participants for a self-determined description of their experiences of discrimination.

### 4.1 Limitations and strengths

This study has a couple of limitations to consider when interpreting the findings. First, the analyses of the 2024 data were cross-sectional and do not allow for the identification of causal explanations for the observed associations. The panel survey is currently being established and will allow for longitudinal analyses in the future. A complex weighting procedure was used to correct for deviations of the sample from official German population statistics for several sociodemographic characteristics, thereby reducing selection bias resulting from systematic non-participation. However, when interpreting the results, it should be noted that translated questionnaires are not yet offered in the panel survey, which would have been important for people who have only recently moved to Germany when deciding whether to participate [93]. Therefore, deviations in the sample are to be expected, for example, in terms of an underrepresentation of people with lower German language proficiency. Additionally, the proportion of people not born in Germany or without German citizenship is lower in the panel survey than would be expected based on the Microcensus [94]. Hence, the prevalence of experiences of discrimination among migrants in the analyses is likely underestimated. Furthermore, this study used interview survey data (i.e. self-reports) and there might be bias (including recall and report bias). Moreover, experiences of discrimination are not necessarily actively perceived, so they may have been underreported. However, the adapted version of the Everyday Discrimination Scale [60, 61] that was used asks for very specific everyday situations, making it less demanding as respondents do not necessarily need to be aware that they have experienced discrimination. It was initially developed to capture racist discrimination and has been used in various studies to analyse associations between discrimination and different health outcomes. Its validity and reliability have been tested in different population groups [61, 95]. Nevertheless, some authors have mentioned that it should be used with caution when applied to diverse categories such as gender, age or education due to its psychometric properties [95, 96], and have encouraged researchers to further develop the instrument.

### 4.2 Conclusion and outlook

This study, which used nationwide data from Germany across different categories of discrimination, showed associations between the frequency of experiences of everyday discrimination, including multiple discrimination, and self-rated general and mental health. Several population groups which experience health inequities more frequently were particularly affected by everyday and multiple discrimination, such as trans and gender diverse people, people in socioeconomically disadvantaged situations and migrants. The WHO has emphasised that it is crucial to overcome and reduce discrimination to promote and foster health equity [4]. Health inequity results from a complex interplay of political, social and economic determinants; structural factors such as living, housing and working conditions; and psychosocial and behavioural factors – which at the same time generate and (re-) produce structural categories relevant for discrimination. Hence, the development of a comprehensive action plan is required to reduce health inequality [4, 97]. According to the health-in-all-policies approach, this mission requires comprehensive and coordinated efforts in many areas of living, society and politics, such as education, labour, social affairs, housing, the environment, healthcare, justice and law enforcement. In this sense, anti-discrimination measures across society as a whole can also be understood as a structural means to prevent health inequality and to promote health. It is essential to strengthen structures for individuals affected by discrimination, also on communal level. That includes the expansion of reporting offices and anti-discrimination registers to make discrimination visible. Additionally, legal reforms are required to enable protection against discrimination in contexts that are not yet (sufficiently) protected by law [5, 98]. Furthermore, issues concerning the criminal relevance of discrimination warrant further discussion [99].

As an outlook for research there is a need for longitudinal data to enable analyses of long-term health-related consequences of discrimination. Additionally, not only interview data should be collected, but also examination data with biological markers to assess the physiological effects of discrimination to better understand the health-related consequences of discrimination. The results from this contribution represent initial basic analyses of a large, population-based sample on associations between discrimination and health for Germany. Results indicate to further deepen analyses, for example with more differentiated statistical methods as are suggested for such research perspectives [42, 44, 100, 101]. In addition, future analyses should focus on more specific health outcomes, health-related behaviours and utilisation of healthcare services regarding everyday discrimination, and discrimination that occurs within the healthcare sector [6, 45, 46]. Overall, the findings underscore that indicators for measuring discrimination should be a constant part of a population-based public health monitoring system in order to capture the importance of discrimination for public health and health equity, track developments over time, and contribute empirically based findings to the discourse on improving health equity.

## Figures and Tables

**Table 1:** Prevalence of experiences of discrimination in everyday life stratified by the demographic, socioeconomic and migration-related characteristics (n = 14,412 women, n = 12,086 men and n = 147 trans/gender diverse people). Source: Panel ‘Health in Germany’ 2024

	Experiences of discrimination in everyday life				
	Rarely/sometimes (n = 18,596)		Often/very often (n = 2,624)		p-value
	%	(95 % CI)	%	(95 % CI)	
**Demographic characteristics**					
**Gender identity**					
Cis female	68.5	(67.5 – 69.6)	12.6	(11.8 – 13.4)	< 0.001
Cis male	69.8	(68.7 – 71.0)	11.7	(10.8 – 12.7)	
Trans/gender diverse	65.4	(54.2 – 75.1)	27.1	(18.6 – 37.8)	
**Age**					
18 – 29 years	66.5	(64.2 – 68.8)	22.8	(20.9 – 24.8)	< 0.001
30 – 44 years	70.1	(68.5 – 71.7)	16.9	(15.4 – 18.4)	
45 – 64 years	72.5	(71.1 – 73.8)	9.9	(9.0 – 10.9)	
65 years and older	65.7	(64.3 – 67.0)	5.0	(4.4 – 5.8)	
**Socioeconomic characteristics**					
**Education**					
Low	65.8	(64.1 – 67.4)	14.5	(13.1 – 16.0)	< 0.001
Medium	70.2	(69.2 – 71.1)	12.5	(11.8 – 13.3)	
High	72.5	(71.3 – 73.7)	7.8	(7.1 – 8.6)	
**Income**					
< 60 % of the median	66.5	(64.3 – 68.5)	18.3	(16.4 – 20.3)	< 0.001
60 – 150 % of the median	69.2	(68.2 – 70.1)	11.5	(10.8 – 12.3)	
> 150 % of the median	71.5	(69.9 – 73.0)	8.9	(7.9 – 10.1)	
**Employment status** ^*^					
Employed	71.7	(70.7 – 72.7)	13.9	(13.1 – 14.8)	< 0.001
Not employed	65.6	(63.3 – 67.9)	18.8	(16.9 – 20.8)	
**Experiences of unemployment within the last five years**					
Yes	65.5	(63.0 – 67.8)	22.4	(20.2 – 24.7)	< 0.001
No	70.1	(69.3 – 70.9)	10.8	(10.2 – 11.4)	
**Migration-related characteristics**					
**Country of birth**					
Germany	69.7	(68.9 – 70.5)	11.5	(10.9 – 12.1)	0.003
Another country	66.3	(63.0 – 69.4)	15.5	(13.1 – 18.2)	
**Citizenship**					
German	69.5	(68.7 – 70.3)	11.7	(11.1 – 12.3)	0.040
Another citizenship	67.5	(63.0 – 71.8)	15.5	(12.4 – 19.2)	
**German language proficiency**					
Native language/very good/good	69.4	(68.6 – 70.2)	11.8	(11.2 – 12.4)	0.007
Moderate/bad/very bad	62.2	(53.7 – 70.0)	20.1	(14.3 – 27.5)	

^*^employable age, 18 – 64 years

CI = confidence interval

**Annex Table 1:** Study population stratified by gender. Source: Panel ‘Health in Germany’ 2024

	Total (n = 26,645)			Cis female (n = 14,412)			Cis male (n = 12,086)		
	n	%	(95 % CI)	n	%	(95 % CI)	n	%	(95 % CI)
**Demographic characteristics**									
**Age**									
18 – 29 years	3,623	15.4	(14.8 – 16.0)	2,084	14.2	(13.4 – 15.0)	1,478	16.3	(15.4 – 17.2)
30 – 44 years	5,685	23.7	(23.0 – 24.4)	3,181	22.7	(21.9 – 23.7)	2,472	24.7	(23.6 – 25.7)
45 – 64 years	8,969	33.7	(33.0 – 34.4)	4,972	33.4	(32.4 – 34.4)	3,975	34.4	(33.4 – 35.4)
65 years and older	8,368	27.2	(26.5 – 27.9)	4,175	29.7	(28.8 – 30.7)	4,161	24.7	(23.7 – 25.6)
**Socioeconomic characteristics**									
**Educational level**									
Low	5,044	33.1	(31.8 – 34.4)	2,446	32.8	(31.4 – 34.2)	2,565	33.2	(31.5 – 35.0)
Medium	12,743	46.4	(45.3 – 47.5)	7,584	48.7	(47.5 – 50.0)	5,079	44.1	(42.6 – 45.5)
High	8,814	20.5	(19.4 – 21.7)	4,351	18.5	(17.4 – 19.6)	4,429	22.7	(21.3 – 24.2)
Missing	44			31			13		
**Income**									
< 60 % of the median	3,882	18.1	(17.4 – 18.9)	2,249	19.4	(18.4 – 20.5)	1,585	16.5	(15.5 – 17.6)
60 – 150 % of the median	16,347	61.6	(60.8 – 62.5)	8,992	62.6	(61.4 – 63.7)	7,276	60.8	(59.6 – 62.0)
> 150 % of the median	6,416	20.2	(19.4 – 21.1)	3,171	18.0	(17.0 – 19.1)	3,225	22.7	(21.5 – 29.9)
**Occupational status** ^*^									
Employed	14,656	79.7	(78.8 – 80.5)	8,002	76.5	(75.3 – 77.7)	6,592	83.0	(81.8 – 84.2)
Not employed	3,608	20.4	(19.5 – 21.2)	2,229	23.5	(22.3 – 24.7)	1,326	17.0	(15.8 – 18.2)
Missing	13			6			7		
**Experiences of unemployment within the last five years**									
Ye s	2,977	14.1	(13.4 – 14.9)	1,663	14.0	(13.1 – 15.0)	1,277	13.9	(12.9 – 15.0)
No	22,959	85.9	(85.1 – 86.6)	12,286	86.0	(85.0 – 87.0)	10,571	86.1	(85.0 – 87.1)
Missing	709			463			238		
**Migration-related characteristics**									
**Country of birth**									
Germany	23,134	89.2	(88.4 – 90.1)	12,492	88.8	(87.7 – 89.8)	10,519	89.7	(88.7 – 90.7)
Another country	1,952	10.8	(9.9 – 11.7)	1,116	11.2	(10.2 – 12.3)	826	10.3	(9.3 – 11.4)
Missing	1,559			804			741		
**Citizenship**									
German	24,183	94.1	(93.4 – 94.7)	13,089	94.0	(93.1 – 94.7)	10,967	94.4	(93.5 – 95.1)
Another citizenship	923	5.9	(5.3 – 6.6)	526	6.0	(5.3 – 6.9)	390	5.6	(4.9 – 6.5)
Missing	1,539			797			729		
**German language proficiency**									
Native language/very good/good	24,801	98.2	(97.8 – 98.4)	13,427	97.9	(97.4 – 98.3)	11,241	98.4	(98.0 – 98.8)
Moderate/poor/very poor	280	1.8	(1.6 – 2.2)	160	2.1	(1.7 – 2.6)	120	1.6	(1.2 – 2.0)
Missing	1,564			825			725		
**Self-reported experiences of discrimination in everyday life**									
Never	5,425	18.6	(18.0 – 19.3)	2,782	18.9	(18.0 – 19.8)	2,633	18.5	(17.6 – 19.5)
Rarely/sometimes	18,596	69.2	(68.4 – 69.9)	10,106	68.5	(67.5 – 69.6)	8,392	69.8	(68.7 – 71.0)
Often/very often	2,624	12.2	(11.6 – 12.9	1,524	12.6	(11.8 – 13.4)	1,061	11.7	(10.8 – 12.7)
**Self-rated general health**									
Very good/good	17,974	64.8	(63.8 – 65.7)	9,669	62.9	(61.8 – 64.0)	8,236	67.0	(65.7 – 68.3)
Fair/poor/very poor	8,648	35.2	(34.3 – 36.2)	4,730	37.1	(36.0 – 38.2)	3,840	33.0	(31.7 – 34.3)
Missing	23			13			10		
**Self-rated mental health**									
Excellent/very good	9,720	34.5	(33.7 – 35.4)	4,596	29.2	(28.2 – 30.3)	5,107	40.4	(39.3 – 41.6)
Good/fair/poor	16,887	65.5	(64.6 – 66.3)	9,798	70.8	(69.7 – 71.8)	6,959	59.6	(58.4 – 60.8)
Missing	38			18			20		

n = number of respondents; % = weighted proportion; CI = confidence interval;

^*^only respondents in employable age (18 – 64 years)

A stratification for trans/gender diverse is not possible reliably due to low number of cases.

**Annex Table 2:** Prevalence ratios for a fair, poor or very poor self-rated general health by the frequency of everyday discrimination and the reported number of reasons (model 3), total and stratified by gender. Source: Panel ‘Health in Germany’ 2024

	Total (n = 24,282)			Cis female (n = 13,076)			Cis male (n = 11,081)		
	PR	(95 % CI)	p-value	PR	(95 % CI)	p-value	PR	(95 % CI)	p-value
**Experiences of discrimination**									
Never (Ref.)	1.00			1.00			1.00		
Rarely/sometimes, 1 reason	**1.15**	(1.08 – 1.23)	< 0.001	**1.12**	(1.03 – 1.23)	0.010	**1.19**	(1.08 – 1.30)	< 0.001
Rarely/sometimes, 2 reasons	**1.39**	(1.29 – 1.50)	< 0.001	**1.34**	(1.22 – 1.48)	< 0.001	**1.43**	(1.28 – 1.60)	< 0.001
Rarely/sometimes, 3 and more reasons	**1.52**	(1.42 – 1.63)	< 0.001	**1.56**	(1.41 – 1.72)	< 0.001	**1.47**	(1.33 – 1.63)	< 0.001
Often/very often, 1 reason	**1.66**	(1.48 – 1.87)	< 0.001	**1.77**	(1.52 – 2.05)	< 0.001	**1.53**	(1.25 – 1.87)	< 0.001
Often/very often, 2 reasons	**1.84**	(1.60 – 2.12)	< 0.001	**1.87**	(1.59 – 2.21)	< 0.001	**1.79**	(1.42 – 2.27)	< 0.001
Often/very often, 3 and more reasons	**2.17**	(1.95 – 2.42)	< 0.001	**2.34**	(2.06 – 2.66)	< 0.001	**1.96**	(1.62 – 2.36)	< 0.001
**Demographic characteristics**									
**Gender identity**									
Cis female	1.03	(0.99 – 1.08)	0.136						
Cis male (Ref.)	1.00								
Trans/gender diverse	**1.62**	(1.26 – 2.07)	< 0.001						
**Age**									
(Metric)	**1.02**	(1.02 – 1.02)	< 0.001	**1.02**	(1.02 – 1.02)	< 0.001	**1.03**	(1.02 – 1.03)	< 0.001
**Socioeconomic characteristics**									
**Educational level**									
Low	**1.17**	(1.11 – 1.24)	< 0.001	**1.20**	(1.12 – 1.29)	< 0.001	**1.17**	(1.07 – 1.27)	0.001
Medium (Ref.)	1.00			1.00			1.00		
High	**0.70**	(0.65 – 0.74)	< 0.001	**0.70**	(0.64 – 0.76)	< 0.001	**0.68**	(0.62 – 0.75)	< 0.001
**Income**									
< 60 % of the median	**1.22**	(1.15 – 1.28)	< 0.001	**1.17**	(1.08 – 1.26)	< 0.001	**1.28**	(1.18 – 1.39)	< 0.001
60 – 150 % of the median (Ref.)	1.00			1.00			1.00		
> 150 % of the median	**0.78**	(0.73 – 0.84)	< 0.001	**0.82**	(0.75 – 0.91)	< 0.001	**0.75**	(0.67 – 0.83)	< 0.001
**Experiences of unemployment within the last five years**									
Yes	**1.53**	(1.43 – 1.63)	< 0.001	**1.50**	(1.37 – 1.65)	< 0.001	**1.55**	(1.40 – 1.71)	< 0.001
No (Ref.)	1.00			1.00			1.00		
**Migration-related characteristics**									
**Country of birth**									
Germany (Ref.)	1.00			1.00			1.00		
Another country	1.05	(0.94 – 1.16)	0.402	1.06	(0.92 – 1.21)	0.417	1.04	(0.89 – 1.21)	0.646
**Citizenship**									
German (Ref.)	1.00			1.00			1.00		
Another citizenship	**0.74**	(0.61 – 0.89)	0.002	**0.73**	(0.58 – 0.93)	0.009	**0.76**	(0.58 – 0.99)	0.043
**German language proficiency**									
Native language/very good/good (Ref.)	1.00			1.00			1.00		
Moderate/poor/very poor	1.25	(0.99 – 1.57)	0.063	**1.44**	(1.08 – 1.93)	0.012	0.99	(0.69 – 1.40)	0.941

PR = adjusted prevalence ratio, CI = confidence interval, Ref. = reference category, bold = significant result (p < 0.05)

**Annex Table 3:** Prevalence ratios for a good, fair or poor self-rated mental health by the frequency of everyday discrimination and the reported number of reasons (model 3), total and stratified by gender. Source: Panel ‘Health in Germany’ 2024

	Total (n = 24,275)			Cis female (n = 13,079)			Cis male (n = 11,071)		
	PR	(95 % CI)	p-value	PR	(95 % CI)	p-value	PR	(95 % CI)	p-value
**Experiences of discrimination**									
Never (Ref.)	1.00			1.00			1.00		
Rarely/sometimes, 1 reason	**1.14**	(1.10 – 1.18)	< 0.001	**1.13**	(1.08 – 1.18)	< 0.001	**1.15**	(1.08 – 1.22)	< 0.001
Rarely/sometimes, 2 reasons	**1.19**	(1.14 – 1.24)	< 0.001	**1.18**	(1.12 – 1.24)	< 0.001	**1.19**	(1.11 – 1.27)	< 0.001
Rarely/sometimes, 3 and more reasons	**1.23**	(1.18 – 1.29)	< 0.001	**1.25**	(1.18 – 1.31)	< 0.001	**1.20**	(1.13 – 1.28)	< 0.001
Often/very often, 1 reason	**1.32**	(1.24 – 1.40)	< 0.001	**1.34**	(1.25 – 1.44)	< 0.001	**1.29**	(1.15 – 1.45)	< 0.001
Often/very often, 2 reasons	**1.45**	(1.37 – 1.55)	< 0.001	**1.46**	(1.38 – 1.55)	< 0.001	**1.42**	(1.25 – 1.62)	< 0.001
Often/very often, 3 and more reasons	**1.43**	(1.35 – 1.52)	< 0.001	**1.37**	(1.28 – 1.47)	< 0.001	**1.52**	(1.38 – 1.68)	< 0.001
**Demographic characteristics**									
**Gender identity**									
Cis female	**1.16**	(1.13 – 1.18)	< 0.001						
Cis male (Ref.)	1.00								
Trans/gender diverse	**1.37**	(1.21 – 1.54)	< 0.001						
**Age**									
(Metric)	**1.00**	(1.00 – 1.01)	< 0.001	**1.00**	(1.00 – 1.00)	< 0.001	**1.01**	(1.01 – 1.01)	< 0.001
**Socioeconomic characteristics**									
**Educational level**									
Low	**1.04**	(1.01 – 1.08)	0.016	1.04	(1.00 – 1.08)	0.070	**1.06**	(1.01 – 1.12)	0.032
Medium (Ref.)	1.00			1.00			1.00		
High	**0.91**	(0.88 – 0.94)	< 0.001	**0.92**	(0.88 – 0.95)	< 0.001	**0.90**	(0.86 – 0.95)	< 0.001
**Income**									
< 60 % of the median	**1.08**	(1.05 – 1.12)	< 0.001	**1.05**	(1.01 – 1.09)	0.011	**1.13**	(1.07 – 1.20)	< 0.001
60 – 150 % of the median (Ref.)	1.00			1.00			1.00		
> 150 % of the median	**0.84**	(0.81 – 0.87)	< 0.001	**0.87**	(0.83 – 0.91)	< 0.001	**0.82**	(0.77 – 0.87)	< 0.001
**Experiences of unemployment within the last five years**									
Yes	**1.14**	(1.10 – 1.18)	< 0.001	**1.10**	(1.05 – 1.14)	< 0.001	**1.18**	(1.11 – 1.25)	< 0.001
No (Ref.)	1.00			1.00			1.00		
**Migration-related characteristics**									
**Country of birth**									
Germany (Ref.)	1.00			1.00			1.00		
Another country	0.98	(0.93 – 1.03)	0.423	1.01	(0.95 – 1.07)	0.792	0.94	(0.85 – 1.04)	0.209
**Citizenship**									
German (Ref.)	1.00			1.00			1.00		
Another citizenship	0.91	(0.83 – 1.01)	0.065	0.90	(0.80 – 1.01)	0.062	0.94	(0.80 – 1.09)	0.394
**German language proficiency**									
Native language/very good/good (Ref.)	1.00			1.00			1.00		
Moderate/poor/very poor	1.08	(0.93 – 1.24)	0.295	1.15	(0.99 – 1.34)	0.063	0.97	(0.75 – 1.24)	0.790

PR = adjusted prevalence ratio, CI = confidence interval, Ref. = reference category, bold = significant result (p < 0.05)
